# Impact of influenza related hospitalization in Spain: characteristics and risk factor of mortality during five influenza seasons (2016 to 2021)

**DOI:** 10.3389/fpubh.2024.1360372

**Published:** 2024-04-02

**Authors:** José-Manuel Ramos-Rincón, Héctor Pinargote-Celorio, Pilar González-de-la-Aleja, José Sánchez-Payá, Sergio Reus, Juan-Carlos Rodríguez-Díaz, Esperanza Merino

**Affiliations:** ^1^Internal Medicine Department, Dr. Balmis General University Hospital, Alicante Institute for Health and Biomedical Research (ISABIAL), Alicante, Spain; ^2^Unit of Infectious Diseases, Dr. Balmis General University Hospital, Alicante Institute for Health and Biomedical Research (ISABIAL), Alicante, Spain; ^3^Preventive Service, Dr. Balmis General University Hospital, Alicante Institute for Health and Biomedical Research (ISABIAL), Alicante, Spain; ^4^Microbiology Service, Dr. Balmis General University Hospital, Alicante Institute for Health and Biomedical Research (ISABIAL), Alicante, Spain

**Keywords:** influenza, epidemics, Spain, hospitalizations, mortality, intensive care unit, economy cost, length of stay

## Abstract

**Background:**

Estimating the global influenza burden in terms of hospitalization and death is important for optimizing prevention policies. Identifying risk factors for mortality allows for the design of strategies tailored to groups at the highest risk. This study aims to (a) describe the clinical characteristics of hospitalizations with a diagnosis of influenza over five flu seasons (2016–2017 to 2020–2021), (b) assess the associated morbidity (hospitalization rates and ICU admissions rate), mortality and cost of influenza hospitalizations in different age groups and (c) analyze the risk factors for mortality.

**Methods:**

This retrospective study included all hospital admissions with a diagnosis of influenza in Spain for five influenza seasons. Data were extracted from the Spanish National Surveillance System for Hospital Data from 1 July 2016 to 30 June 2021. We identified cases coded as having influenza as a primary or secondary diagnosis (International Classification of Diseases, 10th revision, J09-J11). The hospitalization rate was calculated relative to the general population. Independent predictors of mortality were identified using multivariable logistic regression.

**Results:**

Over the five seasons, there were 127,160 hospitalizations with a diagnosis of influenza. The mean influenza hospitalization rate varied from 5/100,000 in 2020–2021 (COVID-19 pandemic) to 92.9/100,000 in 2017–2018. The proportion of influenza hospitalizations with ICU admission was 7.4% and was highest in people aged 40–59 years (13.9%). The case fatality rate was 5.8% overall and 9.4% in those aged 80 years or older. Median length of stay was 5 days (and 6 days in the oldest age group). In the multivariable analysis, independent risk factors for mortality were male sex (odds ratio [OR] 1.14, 95% confidence interval [95% CI] 1.08–1.20), age (<5 years: OR 1; 5–19 years: OR 2.02, 95%CI 1.17–3.49; 20–39 years: OR 4.11, 95% CI 2.67–6.32; 40–59 years: OR 8.15, 95% CI 5.60–11.87; 60–79 years: OR 15.10, 95% CI 10.44–21.84; ≥80 years: OR 33.41, 95% CI 23.10–48.34), neurological disorder (OR 1.97, 95% CI 1.83–2.11), heart failure (OR 1.85, 95% CI 1.74–1.96), chronic kidney disease (OR 1.33, 95% CI 1.25–1.41), chronic liver disease (OR 2.95, 95% CI 2.68–3.27), cancer (OR 1.85, 95% CI 1.48–2.24), coinfection with SARS-CoV2 (OR 3.17, 95% CI 2.34–4.28), influenza pneumonia (OR 1.76, 95% CI 1.66–1.86) and admission to intensive care (OR 7.81, 95% CI 7.31–8.36).

**Conclusion:**

Influenza entails a major public health burden. People aged over 60—and especially those over 80—show the longest hospital stays. Age is also the most significant risk factor for mortality, along with certain associated comorbidities.

## 1 Introduction

In high-income countries, influenza is one of the most important illnesses affecting individuals of all ages. Every year, there are 3 to 5 million severe cases of influenza, of which 240,000 to 650,000 result in death (1, 2). Accurate estimates of the burden of influenza illness on hospital resources are required, both at regional and national levels, in order to allocate hospital resources and determine the cost-effectiveness of specific interventions, including vaccine recommendations (3).

Hospital discharge databases are a valuable source of information to estimate the burden of hospitalization. At least four recent studies in Spain have used the national hospital discharge database, which records information on all hospitalized patients. Specifically, (4) studied inpatient hospital fatality related to influenza from 2009 to 2015; (5), the clinical and economic burden of physician-diagnosed influenza in adults during the 2017–2018 epidemic season; (6), excess hospitalizations and mortality associated with seasonal influenza from 2008 to 2018; and (7), the impact of respiratory syncytial virus and influenza virus infections in adults from 2012 to 2020.

Other European studies have also used hospital discharge databases. In France, there are studies of the characteristics of influenza hospitalizations from the 2012–2013 to 2016–2017 influenza seasons (3) and others estimating the burden of influenza-attributable severe acute respiratory infections on the hospital system in Metropolitan France in different influenza seasons (8, 9). Another study in Portugal investigated excess hospitalizations and mortality associated with seasonal influenza from 2008 to 2018 (10), while in Norway, (11) studied the burden of medically attended influenza from 2008 to 2017.

Estimating the overall burden of influenza in terms of hospitalization, death and economic cost is important for optimizing prevention policies, while identifying risk factors for mortality allows for the design of strategies tailored to vulnerable groups. Moreover, to date, the impact of the COVID-19 pandemic on hospitalizations due to influenza has not been well studied.

In the present study we analyze five influenza seasons in Spain, from 2016–2017 to 2020–2021, aiming to (a) describe the clinical characteristics of hospitalizations with a diagnosis of influenza, (b) assess the associated morbidity (hospitalization rates and ICU admissions rate), mortality and cost of influenza hospitalizations in different age groups and (c) analyze the risk factors for mortality.

## 2 Materials and methods

### 2.1 Data sources

Data on hospital admissions were obtained from the Spanish National Surveillance System for Hospital Data (SNSSHD), specifically the Hospital Care Activity Record - Minimum Basic Data Set (Registro de actividades especializadas-Conjunto Mínimo Básico de datos, RAE-CMBD), which contains all records of hospitalizations in all public and private hospitals in Spain. Diagnoses are coded using the International Classification of Diseases, 10th revision (ICD-10). Data for the Spanish population were obtained from the National Statistics Institute of Spain.

The Spanish Ministry of Health provided data on the annual vaccination coverage in populations over 65 years and dominant circulating influenza virus variants (12). A virus type or subtype was defined as dominant if it accounted for 70% or more of all isolates during the season or if it accounted for 40 to 70%, and the second most common virus accounted for less than 30%. If the second variant accounted for 30% or more, the two types were considered co-dominant (13).

### 2.2 Variables

Hospitalization related to influenza was defined as all recorded hospital admissions with a diagnosis of influenza, defined by ICD-10 codes J09, J10, J11, as either a primary or secondary diagnosis, from 1 July 2016 to 30 June 2021 (five influenza seasons: 2016–2017, 2017–2018, 2018–2019, 2019–2020, 2020–2021). The list of ICD-10 codes used to retrieve data on influenza hospitalization and comorbidities is in Supplementary Table S1.

We included the total number of hospitalizations, stratified by influenza season, age and sex. Age groups were categorized according to 20-year intervals: < 5, 5–19, 20–39, 40–59, 60–79, ≥ 80 years. The hospitalization rate was calculated using the population figures obtained from the National Statistics Institute as of 1 January for each year: 2017 (46.5 million), 2018 (46.9 million), 2019 (46.9 million), 2020 (47.3 million), 2021 (47.4 million).

The variables collected for each hospitalization episode were: age, sex, comorbidities, length of stay (hospitalizations exceeding 60 days were excluded from this analysis), admission to the intensive care unit (ICU), in-hospital mortality and cost. Comorbidities were extracted using ICD-10 diagnostic codes (up to 20 diagnoses, Supplementary Table S1) and classified into the following categories: chronic lung disease, neurological disorders, heart failure, chronic kidney disease, diabetes mellitus, obesity, neoplasia, HIV infection, chronic liver disease and transplantation. The classification of ICU admission encompasses both patients admitted directly to the ICU upon hospitalization and those transferred to the ICU from a different ward after initial admission.

The cost of each episode’s stay is calculated according to the weights and costs of the Diagnosis Related Groups (DRG) patient classification system. This is done based on the clinical-administrative information from the Minimum Basic Data Set and cost data from the Analytical Accounting systems of a sample of hospitals in the system. It is reviewed annually (14).

### 2.3 Analysis

The rates of influenza hospitalization were calculated per 100,000 hospitalizations (obtained from SNSSHD) (15, 16) and per 100,000 population by age group in January 2019. Estimates for the calculation of rates were obtained from the Spanish National Institute of Statistics (17).

Non-parametric continuous variables (as assessed by one-sample Kolmogorov–Smirnov test) are expressed as medians and interquartile ranges (IQR), while categorical variables are expressed as absolute values and percentage. Bivariable comparisons of quantitative and qualitative variables were performed using the Kruskal Wallis test or Mann–Whitney U test for quantitative variables and the Chi^2^ test for qualitative variables. All tests were two-tailed, and only *p* values of less than 0.05 were considered significant. The measure of association was presented as odds ratios (ORs) with their 95% confidence intervals (CIs).

Multivariable logistic regression analysis was used to identify independent predictors of mortality. Variables yielding a p value of less than 0.05 in the crude analysis, plus influenza season to the outcome (in hospital mortality), were entered into a multivariable logistic regression, using a stepwise selection method with the likelihood ratio test. Model discriminatory ability was evaluated using the area under the curve (AUC), a scalar value that represents the overall performance of the model. An AUC of 0.5 suggests no discrimination (similar to random chance), while an AUC of 1.0 indicates perfect discrimination. The regression analysis values were expressed as adjusted ORs and 95% CIs. All statistical analyses were performed using the IBM SPSS package for Windows v25.0 (IBM Corp, Armonk, NY).

### 2.4 Ethical aspects

This study made use of medical data from the SNSSHD. To guarantee patients’ anonymity, the database was provided to us by the Ministry of Health after removing all potential patient identifiers. According to the confidentiality agreement with the Ministry, researchers cannot provide the data to other researchers, so other researchers must request the data directly from the Ministry of Health. The procedures described here were carried out in accordance with the ethical standards described in the Revised Declaration of Helsinki from 2013.

## 3 Results

### 3.1 Incidence and characteristics of hospitalized patients with influenza by age and season

**Figure 1:**
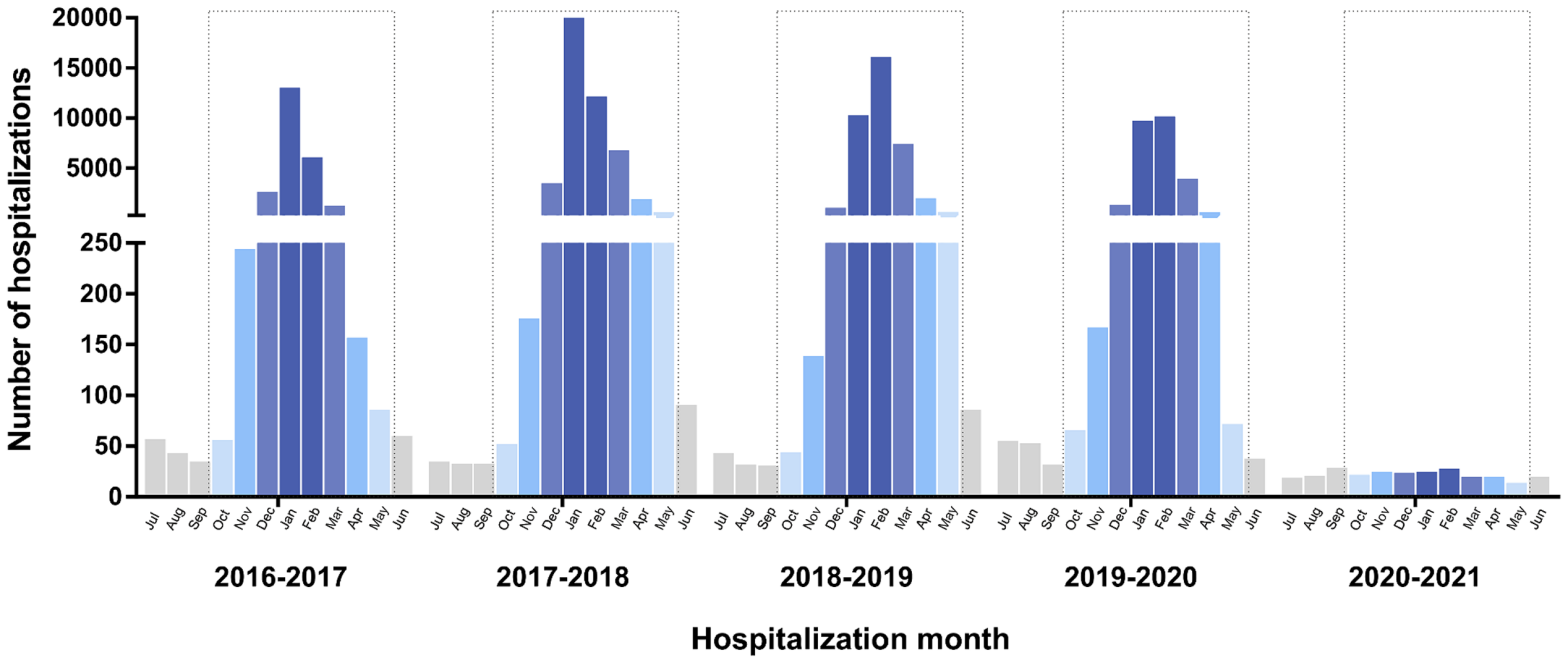
Monthly cases of influenza hospitalization by age group and year, 2016–2021, Spain.

From July 2016 to June 2021, 127,160 hospitalizations with an influenza diagnosis were registered in Spain. The vaccination rate in patients aged 65 years and over remained stable throughout the seasons (mean 61.3%). Figure 1 shows the monthly distribution of cases. Patients aged 60 and over accounted for over 60% of hospitalizations throughout the study period (Table 1). The rate of influenza hospitalization varied from 0.5/100,000 population in 2020–2021 to 92.9 in 2017–2018.

**Table 1:** Dominant influenza virus and vaccination rate in Spain per season and description of influenza hospitalizations per age groups and season, 2016–2021, Spain.

	2016–2017	2017–2018	2018–2019	2019–2020	2020–2021	Total^a^	*p* value^*^
*Epidemiology of influenza in Spain*							
Dominant influenza virus	NA	Influenza B linage not determinate (67%) Influenza A not subtyped (17%) Influenza A (H3) (8%)	Influenza A not subtype (52%) Influenza A (H3) (30%) Influenza A (H1N1) pdm09 (22%)	Influenza A not subtype (43%) Influenza A (H1N1) pdm09 (36%) Influenza B linage not determinate (14%)	Influenza B linage not determinate (75%) Influenza A not subtyped (15%) Influenza A (H1N1) pdm09 (5%)	NA	
Vaccination rate ≥ 65 years	NA	55.9	54.3	66.4	69.5	61.3	
*Influenza hospitalizations*							
Number	22,534	43,596	36,033	24,769	231	127,160	< 0.001
Sex, male, *n* (%)	11,188 (49.7)	22,215 (51.0)	18,320 (50.8)	12,956 (52.3)	137 (59.3)	64,816 (51.0)	< 0.001
Age, median (IQR)	77 (62–85)	74 (58–84)	72 (55–83)	64 (39–79)	65 (43–81)	72 (55–83)	< 0.001
Age-group							
< 5y	1,243 (5.5)	3,120 (7.2)	2,937 (8.2)	3,201 (12.9)	29 (12.6)	10,530 (8.3)	< 0.001
5-19y	530 (2.4)	1,085 (2.5)	1,067 (3.3)	1,383 (5.6)	9 (3.9)	4,074 (3.2)	< 0.001
20-39y	814 (3.6)	1,493 (3.4)	1,571 (4.4)	1705 (6.9)	15 (6.5)	5,598 (4.4)	< 0.001
40-59y	2,367 (10.5)	5,914 (13.6)	5,142 (14.3)	4,524 (18.3)	41 (17.7)	17,988 (14.1)	< 0.001
60-79y	7,475 (33.2)	16,041 (36.8)	12,903 (35.8)	8,184 (33.0)	70 (30.3)	44,673 (35.1)	< 0.001
≥ 80y	10,105 (44.8)	15,940 (26.6)	12,413 (34.4)	5,772 (23.3)	67 (29.0)	44,297 (24.8)	< 0.001

a Average rate per season.

^*^p value was used Chi-square for categorized variables and Kruskal-Wallis test for continuous variables. ^***^J10.9, pneumonia influenza.

**Figure 2:**
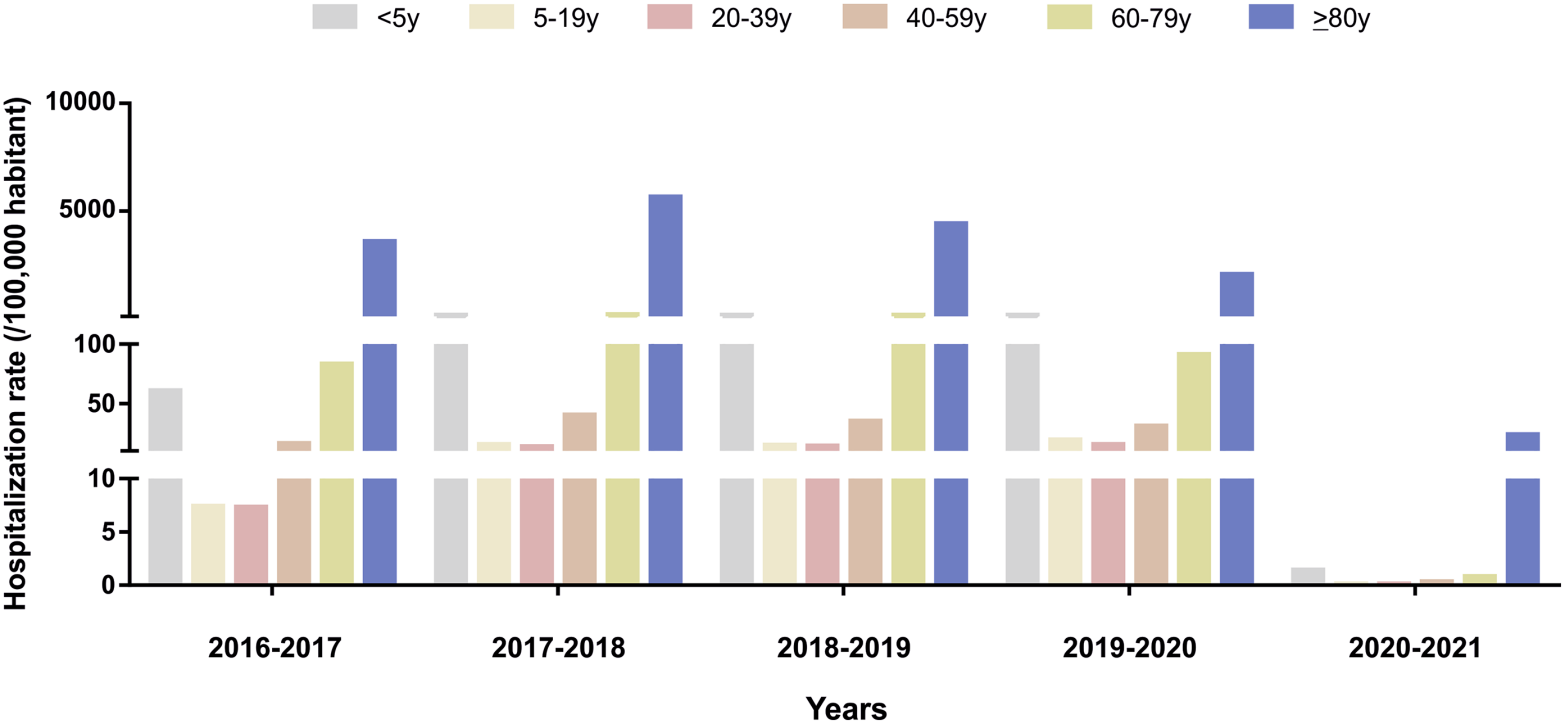
Influenza hospitalization rate (per 100,000 population) in Spain by age group, 2016–2021.

The age distribution of cases varied by season for all hospitalizations (Figure 2; Supplementary Table S2). The highest rates were observed in the oldest age group (≥ 80 years): 3138 hospitalizations per 100,000 population per season, followed by the second-oldest age group (60–79 years: 99 hospitalizations per 100,000 population).

The main comorbidities in hospitalized patients with influenza, across all seasons, were diabetes mellitus (23.8%) and heart failure (15.5%), followed by chronic kidney disease and chronic respiratory disease (Table 2). The admissions rate in people with influenza pneumonia was 15.9% overall and 21.9% in the 2019–2020 season (Table 2).

**Table 2:** Description of influenza hospitalizations epidemiology per season, 2016–2021, Spain.

	2016–2017	2017–2018	2018–2019	2019–2020	2020–2021	Total^a^	*p* value^*^
*Comorbidities*							
Diabetes mellitus	5,701 (25.3)	10,725 (24.6)	8,790 (24.4)	5,052 (20.4)	49 (21.2)	30,317 (23.8)	< 0.001
Heart failure	3,739 (16.6)	6,970 (16.0)	5,785 (16.1)	3,171 (12.8)	41 (17.7)	19,706 (15.5)	< 0.001
Chronic renal failure	3,617 (16.0)	6,661 (15.3)	5,304 (14.7)	2,989 (12.1)	26 (11.3)	18,594 (14.6)	< 0.001
Lung chronic disease	3,451 (15.3)	6,480 (14.9)	5,211 (14.5)	3,120 (12.6)	31 (13.4)	18,293 (14.4)	< 0.001
Obesity	1,995 (8.9)	3,970 (9.1)	3,586 (10.0)	2,408 (9.7)	25 (10.8)	11,984 (9.4)	< 0.001
Neurological disorder	2,052 (9.1)	3,413 (7.8)	2,910 (8.1)	1,331 (5.4)	16 (6.9)	9,722 (7.6)	< 0.001
Neoplasia	780 (3.5)	1,540 (3.4)	1,366 (3.8)	773 (3.1)	7 (3.0)	4,466 (3.5)	0.001
Transplantation	441 (1.8)	864 (2.0)	644 (1.8)	450 (1.8)	6 (2.8)	2,375 (2.6)	0.228
HIV	142 (0.6)	352 (0.8)	250 (0.7)	172 (0.7)	2 (0.9)	918 (0.7)	0.098
Chronic liver disease	159 (0.7)	335 (0.8)	222 (0.6)	154 (0.6)	3 (1.3)	873 (0.7)	0.043
COVID	0 (0.0)	0 (0.0)	0 (0.0)	305 (1.2)	39 (16.9)	344 (0.2)	< 0.001
*Clinical presentation*							
Influenza pneumonia^**^	2,182 (9.7)	6,240 (14.3)	6,394 (17.7)	5,415 (21.9)	30 (13.0)	2,060 (15.9)	< 0.001
*Days of hospitalization*							
Sum	176,260	343,456	280,440	177,029	1833	1,103,467	< 0.001
Median (IQR)	6 (3–10)	5 (3–9)	5 (3–9)	5 (3–9)	5 (2–11)	5 (3–9)	< 0.001
≥ 10 days, *n* (%)	5,591 (25.0)	10,712 (24.8)	8,744 (24.5)	5,295 (21.6)	61 (8.6)	30,403 (24.8)	< 0.001
*Outcome*							
ICU admission	1,373 (6.3)	3,062 (7.0)	2,774 (7.7)	2,076 (8.4)	29 (12.7)	9,314 (7.4)	< 0.001
Mortality	1,417 (6.3)	2,601 (6.0)	2053 (5.7)	1,271 (5.1)	24 (10.4)	7,366 (5.8)	< 0.001

HIV, human immunodeficiency virus; IQR, interquartile range; ICU, intensive care unit.

a Average rate per season.

^*^p value was used Chi-square for categorized variables and Kruskal-Wallis test for continuous variables. ^***^J10.9, pneumonia influenza.

### 3.2 Incidence and characteristics of hospitalized patients with influenza with admission to ICU by Age and season

Admission to the ICU was recorded for 7.4% (n = 9,314) of the total influenza hospitalizations. This proportion was higher during the COVID-19 period (8.4% in 2019–2020 and 12.7% in 2020–2021) (Table 2). The rate of ICU admission varied from 0.6 per 1 million population in 2020–2021 to 65.8 per 1 million population in 2017–2018 (Table 3). The proportion of inpatients with influenza who were admitted to the ICU was highest in those aged 40–59 years (13.9%), with a large gap observed between that age group and the oldest patients (2.5%). Few variations in the proportion of ICU admission were observed within each age group between seasons. In all age groups, this proportion was slighter lower in 2016–2017 and higher in 2020–2021 (which overlapped with second and third wave of COVID-19) (Table 4).

**Table 3:** Rates of influenza hospitalizations, hospitalization with ICU admission and mortality per season, 2016–2021, Spain.

	2016–2017	2017–2018	2018–2019	2019–2020	2020–2021	Total^c^	*p* value^*^
Hospitalization rates (/100,000 population)^a^	48.5	93.5	76.8	52.3	0.5	54.3	< 0.001
Hospitalization rate (/100,000 hospitalization)^b^	512.7	955.4	795.5	543.1	5.7	562.5	< 0.001
Hospitalization rate with ICU admission (/1,000,000 population)^a^	29.5	65.6	59.1	43.9	0.6	39.8	< 0.001
Hospitalization rate with ICU admission (/1,000,000 hospitalization)^a^	312.4	671.1	612.4	455.2	7.2	411.7	< 0.001
Mortality rates (/1,000,000 population)^a^	30.5	55.8	43.8	26.9	0.5	31.5	< 0.001
Mortality rate (/1,000,000 hospitalization)^b^	32.2	57.0	45.3	27.9	0.6	32.6	< 0.001

IQR, interquartile range, ICU, intensive care unit. ^*^p value was used for Kruskal-Wallis test for continuous variables.

a Population size estimates were obtained from the Spanish Institute of Statistics (1st January, 2017, 1,018, 2019, 2020, 2021) for the calculation of rate.

b Hospitalization size were obtained from the Specialized Care Registry - Minimum basic data set (RAE-CMBD) (2016, 2017, 2018, 2019, 2020) for the calculation of rate.

c Average rate per season.

**Table 4:** Frequency of hospitalizations with ICU admission stratified by age and influenza season, 2016–2021, Spain.

	2016–2017			2017–2018			2018–2019			2019–2020			2020–2021			Overall		
Age-group	I	H	I/H (%)	I	H	I/H (%)	I	H	I/H (%)	I	H	I/H (%)	I	H	I/H (%)	I	H	I/H (%)
< 5y	93	1,203	7.7	178	3,111	5.7	188	2,937	6.4	177	3,201	5.5	3	29	10.3	639	10,481	6.1
5-19y	41	505	8.1	84	1,082	7.8	87	1,067	8.2	103	1,383	7.4	1	9	11.1	316	4,046	7.8
20-39y	72	799	9.0	153	1,492	10.3	178	1,571	11.3	176	1705	10.3	1	15	6.7	580	5,582	10.4
40-59y	264	2,290	11.5	816	5,900	13.8	758	5,142	14.7	646	4,524	14.3	9	40	22.5	2,493	17,896	13.9
60-79y	691	7,210	9.6	1,424	16,027	8.9	1,251	12,902	9.7	834	8,184	10.2	11	69	15.9	4,211	44,392	9.5
≥ 80y	212	9,669	2.2	407	15,922	2.6	312	12,413	2.5	140	5,772	2.4	4	67	6.0	1,075	43,843	2.5
Total	1,373	1,203	6.3	3,062	43,534	7.0	2,774	36,032	7.7	2076	24,769	8.4	29	229	12.7	9,314	126,240	7.4

H, Number of hospitalizations; I, Number of hospitalizations with ICU admission.

### 3.3 Days of hospitalization in patients with influenza by age and season

The median length of stay was 5 days (13 days for hospitalizations with ICU admission and 5 days without). This measure increased with age group, from 3 days in patients under 20 years of age to 6 days in those aged 80 years and older, but it was stable within age groups across seasons. The season with highest numbers of hospitalization days was 2017–2018. Altogether, 38.6% of the hospitalization days were in patients aged 60–79 years, followed by 36.6% in the oldest group. In 2016–2017, 82% of all days of hospitalization were in people aged 60 or older (Table 5).

**Table 5:** Hospitalization days for influenza stratified by age and influenza season, 2016–2021, Spain.

	2016–2017	2017–2018	2018–2019	2019–2020	2020–2021	Overall
Age-group	N of days (%)	N of days (%)	N of days (%)	N of days (%)	N of days (%)	N of days (%)
< 5y	5,493 (3.1)	14,077 (4,1)	11,916 (4.2)	12,317 (6.9)	247(13.4)	44,050 (4.5)
5-19y	2,517 (1.4)	5,286 (1.5)	1,063 (0.4)	5,786 (3.2)	35 (1.9)	4,053 (0.4)
20-39y	4,485 (2.5)	9,123 (2.7)	9,539 (3.4)	9,622 (5.4)	42 (2.3)	32,811 (3.4)
40-59y	19,198 (10.9)	49,963 (14.5)	42,798 (15.3)	35,103 (10.8)	339 (18.5)	147,401 (15.1)
60-79y	63,300 (35.9)	135,074 (39.3)	110,786 (39.5)	68,165 (38.5)	554 (30.2)	377,879 (38.6)
≥ 80y	81,267 (46.1)	129,943 (37.8)	100,670 (35.9)	46,036 (26.0)	616. (33.6)	358,532 (36.6)
Total	176,260 (100)	343,466 (100)	280,444 (100)	177,029 (100)	1833 (100)	979,032 (100)

^*^Excluded length of stay > 60 days.

### 3.4 Mortality and risk factor for mortality

Altogether, 5.8% of those hospitalized died. This proportion nearly doubled in 2020–2021 (10.8%). The influenza mortality rate in hospitalized patients ranged from 0.5/1,000,000 population in 2020–2021 to 55.8 in 2017–2018 (Table 3). The case fatality rate increased with age, from 0.3% in children under the age of 5 years to 9.4% in those aged 80 years or older (Table 6). Among the patients admitted to the ICU, the case fatality rate was 19.9% and increased with age, from 3.7% in those aged under 20 years to 29.0% in the oldest patients. People aged 80 and up accounted for 56.6% of total deaths (Table 6).

**Table 6:** Case fatality ratio in hospitalized influenza patients stratified by age and ICU admission, 2016–2021, Spain.

	All hospitalization			ICU admission			Proportion of deaths with ICU admission (%)
Age-group	D	H	CFR	D	H	CFR	
< 5y	29	10,530	0.3	21	639	3.3	72.4
< 5-19y	24	4,074	0.6	14	316	4.4	58.3
20-39y	78	5,598	1.4	50	580	8.6	64.1
40-59y	621	17,988	3.5	374	2,493	15.0	60.2
60-79y	2,440	44,673	5.5	1,082	4,211	25.7	44.3
≥ 80y	4,174	44,297	9.4	312	1,075	29.0	7.5
Total	7,366	127,160	5.8	1853	9,314	19.9	25.2

D: Deaths; H: Hospitalizations; CFR: Case fatality ratio.

Among patients younger than 80 years, 48.3% of deaths occurred after admission to the ICU, compared to 7.5% in the oldest patients (*p* < 0.001). The median interval between hospitalization and death was 8 days (IQR 3–17), and the median length of stay in survivors was 5 days (IQR 3–9) (p < 0.001). The proportion of deaths in each age group was stable across seasons (Table 7).

**Table 7:** Case fatality rate in hospitalized influenza patients stratified by age-group and by season, 2016–2021, Spain.

	2016–2017			2017–2018			2018–2019			2019–2020			2020–2021		
Age-group	D	H	CFR	D	H	CFR	D	H	CFR	D	H	CFR	D	H	CFR
< 5y	3	1,243	0.2	12	3,120	0.4	5	2,937	0.2	7	3,201	0.2	2	29	6.9
5-19y	6	530	1.1	4	1,085	0.4	6	1,067	0.6	8	1,383	0.6	0	9	0.0
20-39y	6	814	0.7	21	1,493	1.4	27	1,571	1.7	24	1705	1.4	0	15	0.0
40-59y	82	2,367	3.5	211	5,914	3.6	179	5,142	3.5	147	4,524	3.2	2	41	4.9
60-79y	365	7,475	4.9	869	16,041	5.4	693	12,903	5.4	504	8,184	6.2	9	70	12.9
≥ 80y	955	10,105	9.5	1,484	15,940	9.3	1,143	12,413	9.2	581	5,772	10.1	11	67	16.4
Total	1,417	22,534	6.3	2,601	43,593	6.0	2053	36,033	5.7	1,271	24,769	5.1	24	231	10.4

D: Deaths; H: Hospitalizations; CFR: Case fatality ratio.

**Figure 3:**
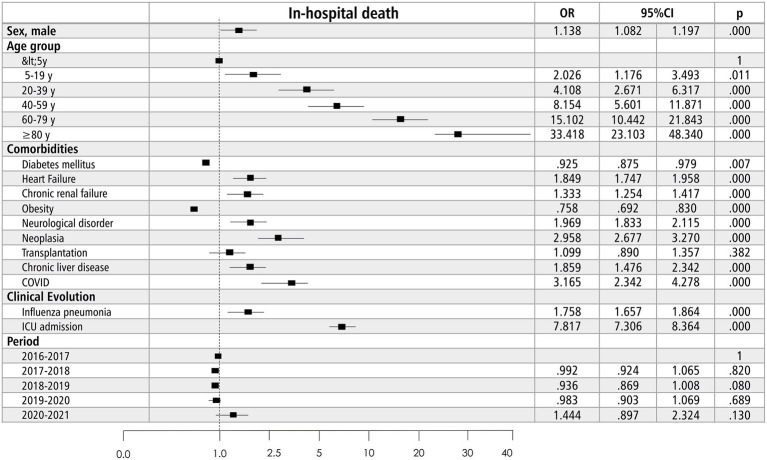
Risk factors of in-hospital death due to influenza in Spain, 2016–2021.

Table 8 compares the epidemiological and clinical characteristics in people who survived versus died during their influenza hospitalization, and Supplementary Table S3 presents a more specific comparison by season. In the multivariable analysis (Figure 3), age (<5 years: OR 1; 5–19 years: OR 2.02, 95%CI 1.17–3.49; 20–39 years: OR 4.11, 95% CI 2.67–6.32; 40–59 years: OR 8.15, 95% CI 5.60–11.87; 60–79 years: OR 15.10, 95% CI 10.44–21.84; ≥80 years: OR 33.41, 95% CI 23.10–48.34). Other independent risk factors were male sex (OR 1.14, 95% CI 1.08–1.20), neurological disorder (OR 1.97, 95% CI 1.83–2.11), heart failure (OR 1.85, 95% CI 1.74–1.96), chronic kidney disease (OR 1.33, 95% CI 1.25–1.41), chronic liver disease (OR 2.95, 95% CI 2.68–3.27), cancer (OR 1.85, 95% CI 1.48–2.24), coinfection with SARS-CoV2 (OR 3.17, 95% CI 2.34–4.28), influenza pneumonia (OR 1.76, 95% CI 1.66–1.86) and admission to intensive care (OR 7.81; 95% CI 7.31–8.36). Obesity was a protective factor (OR 0.76, 95% CI 0.69–0.83). The AUC was 0.79 (95% CI 78–0.79, *p* < 0.001). No differences in risk factors were observed between the different flu seasons.

**Table 8:** Risks factor of death of influenza hospitalizations, 2016–2021, Spain.

Variable	Survival *N* (%)	Death *N* (%)	*p* value
Sex, male	60,979 (94.1)	3,837 (5.9)	0.048
*Age group*			
< 5	10,501 (99.7)	29 (0.4)	1
5–19 y	4,050 (99.4)	24 (0.6)	0.006
20-39y	5,520 (98.6)	78 (1.4)	< 0.001
40-59y	17,367 (96.5)	621 (3.5)	< 0.001
60-79y	42,233 (94.5)	2,440 (5.5)	< 0.001
≥ 80y	40,123 (90.6)	4,174 (9.4)	< 0.001
*Comorbidities*			
Diabetes mellitus	28,242 (93.2)	2074 (6.8)	< 0.001
Heart Failure	17,366 (88.1)	2,340 (11.9)	< 0.001
Chronic renal failure	16,792 (90.3)	1802 (9.7)	< 0.001
Lung chronic disease	17,180 (93.9)	1,113 (6.1)	0.068
Obesity	11,387 (95.0)	587 (5.0)	< 0.001
Neurological disorder	8,560 (88.0)	1,162 (12.0)	< 0.001
Neoplasia	3,919 (87.8)	547 (12.2)	< 0.001
Transplantation	2,269 (95.5)	106 (4.5)	0.005
Chronic liver disease	779 (89.2)	94 (10.8)	< 0.001
HIV	878 (95.6)	40 (4.4)	0.062
COVID	344 (84.1)	65 (15.9)	< 0.001
*Clinical evolution*			
Influenza pneumonia*	18,298 (90.3)	1962 (9.7)	< 0.001
ICU admission	7,461 (80.1)	1853 (19.9)	< 0.001
*Influenza season*			
2016–2017	21,117 (93.7)	1,417 (6.3)	1
2017–2018	40,992 (94.0)	2,601 (6.0)	0.101
2018–2019	33,980 (94.3)	2053 (5.7)	0.003
2019–2020	23,498 (94.9)	1,271 (5.1)	< 0.001
2020–2021	207 (89.6)	24 (10.4)	0.012

ICU, intensive care unit. ^*^J10.9, pneumonia influenza.

### 3.5 Economic burden of influenza

**Figure 4:**
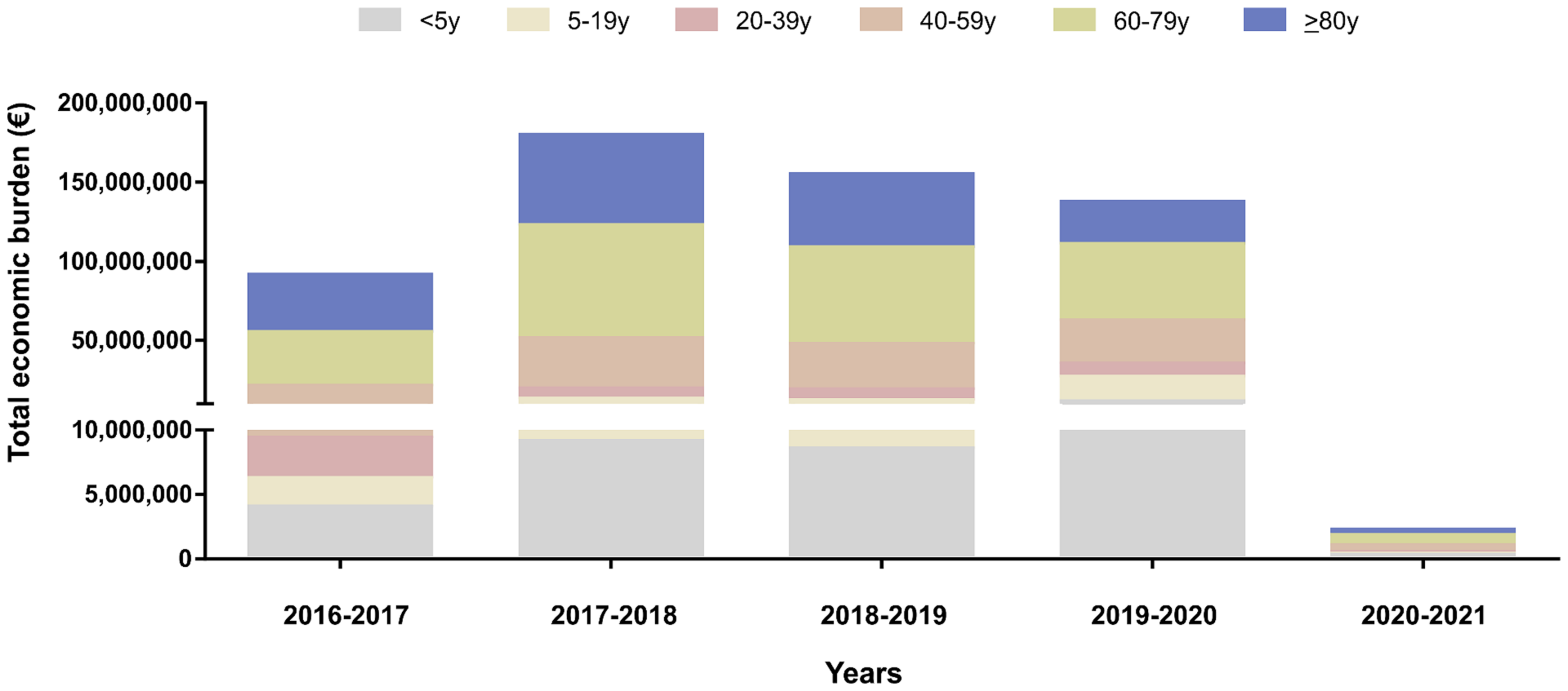
Total economic burden of influenza hospitalization in Spain, by age group and year, 2016–2021.

Over five influenza seasons, the direct costs of 127,160 hospitalizations for influenza in the Spanish NHS were an estimated EUR 554.6 million. This figure varied considerably by epidemic season, from EUR 2.2 million in 2020–2021 during the COVID-19 pandemic, with only 231 cases, to EUR 179.5 million in 2017–2018. The age groups incurring the highest costs were 60–79 years old (EUR 215.5 million, 38.9% of the total) and ≥ 80 years (EUR 165.9 million, 29.9%), with similar proportions across all influenza seasons (Figure 4). The median cost per hospitalization steadily increased from EUR 3252 in 2016–2017 to EUR 4079 in 2020–2021 (Supplementary Table S7).

## 4 Discussion

This study described the characteristics and severity of influenza hospitalizations over five epidemic seasons, as coded using the ICD-10 in the Spanish national hospital database. There were an estimated 20,000 to 40,000 annual hospital admissions and one million days of hospital stay during this period, with significant variation between seasons. Notably, there was a progressive decrease (of almost a decade) in the median age of hospitalized patients over the years. Severity criteria (ICU admission, mortality and length of stay) were associated with age and remained stable throughout different seasons. The main risk factor for mortality was age, doubling the risk every 20 years. Another risk factor was the development of influenza pneumonia and certain comorbidities, such as cancer, heart failure and chronic neurological, kidney, or liver disease.

In the context of the COVID-19 pandemic (season 2020–2021), there was a dramatic reduction in hospital admissions due to influenza but also an increased proportion who needed ICU admission (12.7% versus 6.3–8.4% in non-pandemic years). Hospitalized patients also showed a lower median age and a higher mortality rate compared to previous influenza seasons, which can likely be attributed to the decrease in admissions of patients with mild–moderate illness. Moreover, during the COVID-19 pandemic, the incidence of other viral respiratory diseases, including influenza and respiratory syncytial virus, decreased dramatically (for example in a study in Portugal from March to April 2021), the Cobas Influenza/RSV assay was used to test 2,132 samples from children and adults presenting to the emergency department, and all were negative for RSV and influenza (18). In another hospital-based study in Madrid (Spain) during the 2020–2021 season, flu circulation likewise disappeared, but influenza A reappeared in November 2021, reaching a maximum peak detection in April 2022, and this was reflected in the hospitalization rates (19). In the 2020–2021 season, only 231 patients were admitted with an influenza diagnosis in Spain, a 106-fold reduction relative to the preceding flu season. Similarly, mobility restrictions or population quarantine during the COVID-19 pandemic contributed to a lower incidence of flu infections, and therefore, lower hospitalizations and associated costs, as demonstrated in studies during and after COVID-related border restrictions (20).

Influenza caused a mean 54.2 hospitalizations per 100,000 population (range 0.5 to 92.9 per season) from 2016–2017 to 2020–2021. Compared to European reports based on similar administrative databases, our observed rates are higher than in France (28.5 hospitalizations per 100,000 population from 2012 to 2017 (3)) and Portugal (11.6 cases per 100,000 population from 2008 to 2018 (10)), but similar to those published in Norway (48 hospitalizations per 100,000 population from 2008 to 2017 (11)) and the UK (49 per 100,000 population from 1997 to 2009) (21).

However, none of these reports included the 2020–2021 season, coinciding with the start of the COVID-19 pandemic. If we exclude this outlier, the hospitalization rate in Spain from 2016–2017 to 2019–2020 was 67.6 per 100,000 population, significantly higher than in the rest of European countries with available data. In other Spanish reports based on hospital discharge databases, the hospitalization rate varies between seasons, ranging from 1804 cases per 100,000 population in individuals aged 18 years or older in the 2017–2018 season [5], to 9.7 in 2009–2015, 28.1 from 2008–2009 to 2017–2018 (6) and 9.7 in 2009–2015 (4). These differences can be explained by the heterogeneity of the population pyramid, hospitalization criteria and healthcare systems in each country. But the influence of circulating virus variants, vaccination rates, and the effectiveness of vaccination may be the main explanations for the different hospitalization rates (22), which would also explain the difference in our analysis across the four seasons prior to COVID-19, with rates ranging from 48 to 92.9 per 100,000 population. In this regard, the rate in the 2017–18 season was double that of other seasons.

Our data confirm the influence of age on influenza hospitalization, as reported in the literature (3, 10, 11, 21), with a marked increase in admission rates in adults aged 60–79 years and especially in those aged 80 years or older (from 99.3 to 3,138 per 100,000 population). However, these rates were higher than those recently published in other countries. In Norway, the hospitalization rate in those aged 80 years or older was 825 per 100,000 population during the 2016–17 season (11), and in the UK the rate in people aged 75 years or older was 329 hospitalizations per 100,000 population from 1997–2009 (21).

The reduction in the median age of hospitalized patients over the years is also notable, dropping from 77 in 2016–2017 to 65 years in 2020–21. This trend may be related to the progressively increasing hospitalizations in children under 5 years old (whose proportion doubled relative to total influenza hospitalizations over the study period), the higher vaccination rate in patients over 65 years old, or the impact of the pandemic, which could have reduced hospitalizations in patients over 80 years old.

The median length of hospital stay was stable over the study period at around 5 days. Overall, influenza was implicated in approximately one million days of hospital stay in Spain, equating to around 200,000 to 250,000 hospital-days per season. These data allow for the estimation of healthcare resources required for each flu season. Additionally, they facilitate the assessment of vaccination effectiveness, not only in terms of infection incidence or mortality but also in reducing the length of hospital stay.

The proportion of patients admitted to the ICU was lower among old (60–79 years) and very old patients (≥ 80 years). This finding does not reflect the severity of the flu infection, since most deaths in very old people occurred outside the ICU (92%), as also reported by Pivette et al. (81% of deaths in ≥80 years were outside the ICU). Rather, the comorbidities, frailty, and other baseline conditions in this group probably led physicians to limit therapeutic efforts. Based on similar data, (3) proposes including other data beyond ICU admission when assessing the severity of hospitalization in very old patients.

The overall mortality rate among hospitalized patients remained stable at around 6%, except in 2020–2021, when it reached 11%. In that pandemic season, hospitalized patients were younger and showed a higher proportion of ICU admissions. However, the primary factor that may contribute to the strikingly high mortality rate is the selective hospitalization of patients with severe influenza, given the healthcare overload during the pandemic. We do not have data on vaccination rates in this population, but this result could also be related to the lower rate compared to older patients, in whom vaccination is universally recommended.

Despite the differences between seasons, populations and healthcare systems, the case fatality rate reported in hospitalized patients is similar to ours in most cohorts reported in the literature. In France, the overall mortality rate was 5.8% (95% CI 5.6–6.0%) in 45,819 patients admitted during the 2018–2019 season (23). In a German cohort of 6,762 patients from 2017 to 2019, the overall mortality was 6% (95% CI 5.5–6.6%) (24). Lastly, an analysis conducted in 18 countries during the 2018–2019 season, evaluating 3,512 patients, showed a mortality rate of 6.1% (95% CI 5.3–6.9%) (25). In contrast, another study in Spain from 2010 to 2016 reported an overall mortality rate of 13% (95% CI 11.5–14.7%) among 1726 hospitalized patients with influenza (26). Three cohorts showed lower mortality than in our study: the US veterans cohort showed a mortality rate of 3.2% (95% CI 2.5–3.9%) in hospitalized patients in 2022–23 (27), similar to the 3% (95% CI 2.4–3.8%) reported in Norway from 2008 to 2017 (11) and slightly lower than the 4.9% (95% CI 4.8–5.1%) reported in France from 2012 to 2017 (3). As with the hospitalization rates, the same factors may be responsible for the different mortality rates (population, vaccination, disease severity, healthcare systems), plus differences in the prescribed therapies for influenza.

Our data show an age-related increase in mortality, with the case fatality rate rising from 0.3 in people under of 5 years to 9.4 in those aged 80 years and older. Indeed, over half the deaths were in the oldest age group. This finding is consistent with most other published cohorts. The overall mortality in one French cohort increased from to 5.8 to 13.8% in those over 90 years old (23), while in a Dutch cohort, rates ranged from 6 to 9% in people aged 70–89 years, compared to 14% in those over 90 (24). The (3) cohort also showed an increase in case fatality according to age, from 4% in those aged 40–59 years to 7% in those aged 60–79 years and 10% in people over 80 years old. Another Dutch study, this one taking place from the 2011–2012 to 2019–2020 seasons, estimated a 20% increased risk of a fatal outcome with each decade of life, indicating a doubling of risk every 5 years of age (28).

Regarding risk factors associated with mortality, age was the most important, consistent with previous reports (5, 6, 25, 26). According to our data, the risk of mortality doubled with every 20 years of advancing age, with people over 80 showing a 26-fold higher risk than those under 20. Previous studies have also identified comorbidity as a risk factor for mortality. In an analysis conducted in 18 countries using administrative databases with coding rules, (24) reported that age, diabetes and chronic obstructive pulmonary disease were associated with mortality, while male sex and influenza A infection were protective. Other clinical reports identify male sex, immunosuppression, obesity, bacterial co-infection and ICU admission as additional risk factors (26).

Our analysis confirms an independent increase in mortality in patients with neurological disorders, heart disease, chronic kidney disease, chronic liver disease and cancer. Notably, neither obesity, diabetes nor transplantation were significant risk factors in our cohort. While obesity has been linked to increased mortality (29), particularly during the H1N1 influenza pandemic, it may not be adequately represented in the databases (under-coding of obesity in a national hospitalization database). However, several hypotheses support a diminished mortality risk in obese people, which may be insufficiently captured in the databases. One hypothesis proposes that vaccination initiatives aimed at high-risk or obese cohorts, including those with severe obesity, have attenuated mortality risk. Another hypothesis suggests that adjusting for underweight status abolishes the impact of obesity.

The absence of any association with diabetes is currently unexplained, but it could be related to a higher vaccination rate, as discussed elsewhere (30). As for organ transplantation, this variable may have low prevalence in the overall cohort, leaving the analysis underpowered. The presence of influenza-related pneumonia or ICU admission, as clinical manifestations of severity, was also associated with mortality in our analysis; however, we did not identify an impact across different seasons. Finally, viral variants, vaccination coverage, bacterial coinfection and treatment variables were not included in the databases, so their relationship with mortality could not be assessed.

Regarding the economic impact of influenza, the mean annual cost of these hospitalizations, after excluding the 2020–2021 season, was EUR 127 million. Previous studies in Spain have associated the scale of these costs with age and the comorbidity burden (5, 6), using two methods to calculate the economic cost of influenza in hospital care (BARI study). They reported that about two-thirds of the mean direct annual costs of hospitalizations with a primary or secondary diagnosis of influenza, which totaled EUR 45.7 million, were generated by patients with comorbidities. In their estimation using broader diagnostic groups and time series models, the mean direct annual cost of the all excess cardiovascular and respiratory hospitalizations that were related to influenza ascended to EUR 142.9 million across age groups and EUR 115.9 million in patients aged 65 years and up. For their part, (5) estimated the mean healthcare cost per case at EUR 235.1 in people aged 18–49 years, with costs increasing 1.7- and 4.9-fold in older age groups (50–64 years, EUR 402; ≥ 65 years, EUR 1149).

### 4.1 Strengths and limitations

Our study has several strengths. First, we used a medical-administrative database with coding rules that included all influenza-related hospitalizations across Spain over a five-season period. Second, to our knowledge, our study is the first to analyze influenza hospitalization and mortality during the COVID-19 pandemic. Third, the study took a multidisciplinary approach, with epidemiological data (hospitalization incidence, age stratification), clinical data (length of stay, ICU admission, risk factors for mortality) and economic data.

On the other hand, limitations include those typically associated with the use of medico-administrative databases with coding rules. First, there are some risks of bias, related to: underreporting (all public hospitals must report all patients admitted to hospital; however, private hospitals are not required to do so), severity (the most severe cases are those that are admitted, entailing an overrepresentation of mortality), diagnostic accuracy (the diagnosis of influenza can be clinical or virological by RT-PCR, and clinically diagnosed cases may be other respiratory viral infections), healthcare utilization pattern (more than 90% of the Spanish population exclusively uses the public healthcare scheme, but patients with insurance can use a public hospital if they came from emergency department), differences in healthcare practices between hospitals (for instance, their admission criteria). However, we considered that our definition of influenza hospitalization was very specific, as we assumed that the attribution of an ICD-10 influenza code relies on a viral confirmation test as per guideline recommendations. However, the SNSSHD used in this study, which has been validated for influenza infection in Spain (31), showed 79.87% sensitivity, 99.72% specificity, 86.71% positive predictive value and 99.54% negative predictive.

Moreover, we have incorporated ICD-10 codes pertaining to influenza as either a primary or secondary diagnosis. Notably, instances where influenza is classified as a secondary diagnosis may result in an overestimation of metrics such as length of hospital stay, admission to the intensive care unit (ICU), mortality rates, and associated costs, in comparison to cases where it is designated as a primary diagnosis. However, it is plausible that in such instances, the admission for influenza exacerbates the underlying conditions that were deemed more clinically significant by healthcare professionals, thereby warranting the classification of influenza as a secondary diagnosis. Additionally, the number of deaths related to influenza are underestimated because we do not include deaths occurring outside the hospital (death certificates). Furthermore, this was a retrospective study, so we had no opportunity to review patients’ medical histories, which would have allowed us to check data for accuracy (quality of information on causes of death). Finally, we do not have vaccination coverage data for the total population, which can determine hospitalization rates and mortality incidence.

## 5 Conclusion

In conclusion, the overall impact of influenza hospitalization over five seasons, 2016–2017 to 2020–2021, resulted in over a million days of hospital admission, with significant variability in the number of admissions according to the season analyzed, with hospitalizations in some seasons doubling those in others. The age group with the longest hospital stay and highest mortality is the population over 60 years old and especially those over 80 years. The most significant risk factors for mortality are age and certain associated comorbidities. The COVID-19 pandemic was associated with much lower rates of hospital admissions, but with younger patients and higher mortality.

The influenza hospitalizations recorded in the SNSSHD give key information on the burden of severe influenza in Spain. The present study highlights the major public health burden of influenza and its severe complications. Annual analyses of these data are valuable in order to document the severity of influenza hospitalizations by age group and influenza variants in circulation. Improvements in prevention and treatment strategies, especially in the population at higher risk, could reduce the disease burden associated with influenza as well as healthcare costs.

## Data availability statement

Publicly available datasets were analyzed in this study. This data can be found here: The datasets generated during the current study are not publicly available due data are not publicly available due to privacy or ethical restrictions, but are available from the corresponding author on reasonable request.

## Author contributions

J-MR-R: Writing – review & editing, Writing – original draft, Validation, Methodology, Formal analysis, Conceptualization. HP-C: Writing – review & editing, Methodology, Formal analysis. PG-d-l-A: Writing – review & editing, Validation, Methodology. JS-P: Writing – review & editing, Formal analysis. SR: Writing – review & editing, Methodology. J-CR-D: Writing – review & editing, Validation, Methodology. EM: Writing – review & editing, Writing – original draft, Supervision, Methodology, Conceptualization.
