# Supplementary material for: Impact of influenza related hospitalization in Spain: characteristics and risk factor of mortality during five influenza seasons (2016 to 2021)
Source: Front Public Health. 2024 Apr 2;12:1360372. doi: 10.3389/fpubh.2024.1360372 (PMC11018950; doi:10.3389/fpubh.2024.1360372)
Supplement: Supplementary file 1 [file Table_1.DOCX]

**Table 1S. List of ICD 10-M Codes used in the study**

| **Disease** | **ICD 10-M** |
| --- | --- |
| Influenza | J09, J10, J11 |
| Influenza pneumonia | J10.0 |
| **Comorbidities** |  |
| Heart failure | I50 |
| Dementia and cerebrovascular disease | F01, F02,F03, G30,G31, G46, G45 |
| Chronic obstructive pulmonary disease | J45 |
| Solid and hematological malignancy | C00–C80 and C81– C96 |
| Diabetes mellitus | E11, E13 |
| Chronic kidney disease | N18 |
| Transplantation | Z94 |
| Obesity | E66 |
| Chronic liver disease | K73, K74 |
| HIV infection | B20 |
| COVID- 10 | U07.1, B34.2, B97.29, J12.82, J12.81 |
