# Supplementary material for: Impact of influenza related hospitalization in Spain: characteristics and risk factor of mortality during five influenza seasons (2016 to 2021)
Source: Front Public Health. 2024 Apr 2;12:1360372. doi: 10.3389/fpubh.2024.1360372 (PMC11018950; doi:10.3389/fpubh.2024.1360372)
Supplement: Supplementary file 2 [file Table_2.DOCX]

**Table 2S. Hospitalization rate (/100,000 habitants) of influenza per age‐group and per year, 2016‐2021, Spain**

| **Age‐group** | **2016-2017** | **2017-2018** | **2018-2019** | **2019-2020** | **2020-2021** | **Total** |
| --- | --- | --- | --- | --- | --- | --- |
| < 5y | 60.6 | 152.1 | 143.1 | 156.0 | 1.4 | 102.6 |
| 5-19y | 7.4 | 15.2 | 14.9 | 19.3 | 0.1 | 11.4 |
| 20-39y | 7.3 | 13.4 | 14.1 | 15.3 | 0.1 | 10.1 |
| 40-59y | 16.1 | 40.2 | 34.9 | 30.7 | 0.3 | 24.4 |
| 60-79y | 83.1 | 178.4 | 143.5 | 91.0 | 0.8 | 99.3 |
| ≥ 80y | 3579.4 | 5646.2 | 4396.9 | 2044.5 | 23.7 | 3138.2 |
