# Supplementary material for: Impact of influenza related hospitalization in Spain: characteristics and risk factor of mortality during five influenza seasons (2016 to 2021)
Source: Front Public Health. 2024 Apr 2;12:1360372. doi: 10.3389/fpubh.2024.1360372 (PMC11018950; doi:10.3389/fpubh.2024.1360372)
Supplement: Supplementary file 3 [file Table_3.DOCX]

**Table 3S. Risks factor of mortality of influenza hospitalizations by season, 2016‐2021, Spain**

|  | **2016-2017** | | | **2017-2018** | | | **2018-2019** | | | **2019-2020** |  |  | **2020-2021** |  |  |
| --- | --- | --- | --- | --- | --- | --- | --- | --- | --- | --- | --- | --- | --- | --- | --- |
| **Variable** | **Survival**  **N (%)** | **Death**  **N (%)** | **P value** | **Survival**  **N (%)** | **Death**  **N (%)** | **P value** | **Survival**  **N (%)** | **Death**  **N (%)** | **P value** | **Survival**  **N (%)** | **Death**  **N (%)** | **P value** | **Survival**  **N (%)** | **Death**  **N (%)** | **P value** |
| Sex, male | 10500 (49.7) | 688 (48.6) | 0.392 | 20848 (50.9) | 1367 (52.6) | 0.093 | 16742 (49.3) | 971 (47.3) | 0.082 | 12271 (52.2) | 685  (53.9) | 0.245 | 122  (58.9) | 15 (62.5) | 0.737 |
| **Age group** |  |  |  |  |  |  |  |  |  |  |  |  |  |  |  |
| < 5 | 1240 (5.9) | 3  (0.2) | 1 | 3108 (7.6) | 12 (0.5) | 1 | 2932 (8.6) | 5  (0.2) | 1 | 3194  (13.6) | 7  (0.6) | 1 | 27  (13.0) | 2  (8.3) | 1 |
| 5-19y | 534 (2.5) | 6  (0.4) | 0.028 | 1081 (2,6) | 4 (0.29 | 0.941 | 1061 (3.1) | 6  (0.3) | 0.048 | 1375  (5.9) | 8  (0.6) | 0.060 | 9  (4.3) | 0  (0.0) | NA |
| 20-39y | 808 (3.8) | 6  (0.4) | 0.113 | 1472 (3.6) | 21 (0.8) | <0.001 | 1544 (4.5) | 27  (1.3) | <0.001 | 1681  (7.2) | 24  (1.9) | <0.001 | 15  (7.2) | 0  (0.0) | NA |
| 40-59y | 2285 (10.8) | 82  (5.8) | <0.001 | 5703 (13.9) | 21 (8.1) | <0.001 | 4963 (14.6) | 179  (8.7) | <0.001 | 4377  (18.6) | 147  (11.6) | <0.001 | 39  (18.8) | 2  (8.3) | 0.721 |
| 60-79y | 7710 (33.7) | 365 (25.8) | <0.001 | 15172 (37.0) | 869 (33.4) | <0.001 | 12210 (35.9) | 693 (33.8) | <0.001 | 7680  (32.7) | 504  (39.7) | <0.001 | 61  (29.5) | 9  (37.5) | 0.398 |
| ≥ 80y | 9150 (43.3) | 955 (67.4) | <0.001 | 14456 (35.3) | 1484 (57.1) | <0.001 | 11270 (33.2) | 1143 (55.7) | <0.001 | 5191  (22.1) | 581  (45.7) | <0.001 | 56  (27.1) | 11 (45.8) | 0.225 |
| **Comorbidities** |  |  |  |  |  |  |  |  |  |  |  |  |  |  |  |
| Diabetes mellitus | 5297 (25.1) | 404 (28.5) | 0.004 | 10005 (24.4) | 720 (27.7) | <0.001 | 8214 (24.2) | 576 (28.1) | <0.001 | 4682  (19.9) | 370  (29.1) | <0.001 | 44  (21.3) | 5  (20.8) | 0.962 |
| Heart Failure | 3293 (15.6) | 446 (31.5) | <0.001 | 6145 (15.0) | 825 (31.7) | <0.001 | 5111 (15.0) | 674 (32.8) | <0.001 | 2784  (11.8) | 387  (20.4) | <0.001 | 33  (15.9) | 8  (33.3) | 0.035 |
| Chronic renal failure | 3253 (15.4) | 361 (25.5) | <0.001 | 6025 (14.7) | 626 (24.1) | <0.001 | 4770 (14.0) | 534 (26.0) | <0.001 | 2713  (11.5) | 276  (21.7) | <0.001 | 21  (10.1) | 5  (20.8) | 0.162 |
| Lung chronic disease | 3215 (15.29) | 236 (16.7) | 0.148 | 6101 (14.9) | 379 (14.6) | 0.0664 | 4907 (14.4) | 304 (14.8) | 0.646 | 2931  (12.5) | 189  (14.9) | 0.012 | 26  (12.6) | 5  (20.8) | 0.260 |
| Obesity | 1888 (8.9) | 107 (7.6) | 0.075 | 3778 (9.2) | 192 (7.4) | 0.002 | 3220 (10.1) | 166  (8.1) | 0.004 | 2278  (9.7) | 130  (10.2) | 0.532 | 23  (11.1) | 2  (8.3) | 0.999 |
| Neurological disorder | 1796 (8.5) | 256 (18.1) | <0.001 | 3017 (7.4) | 396 (15.2) | <0.001 | 2568 (7.6) | 342 (16.7) | <0.001 | 1167  (5.0) | 164  (12.9) | <0.001 | 12  (5.8) | 4  (16.7) | 0.047 |
| Neoplasia | 699 (3.3) | 81  (5.7) | <0.001 | 1349 (3.3) | 191 (7.3) | <0.001 | 1195 (3.5) | 171  (8.3) | <0.001 | 671  (2.9) | 102  (8.0) | <0.001 | 5  (2.4) | 2  (8.3) | 0.109 |
| Transplantation | 396 (1.9) | 15  (1.1) | 0.026 | 820 (2.0) | 44 (1.7) | 0.191 | 615 (1.8) | 29  (1.4) | 0.187 | 433  (1.8) | 17  (1.3) | 0.189 | 5  (2.4) | 1  (4.2) | 0.486 |
| HIV | 134 (0.6) | 8  (0.6) | 0.747 | 339 (0.8) | 13 (0.5) | 0.071 | 238 (0.7) | 12  (0.6) | 0.539 | 165  (0.7) | 7  (0.6) | 0.527 | 2  (1.09) | 0  (0.0) | 0.999 |
| Chronic liver disease | 142 (0.7) | 17  (1.2) | 0.022 | 302 (0.7) | 33 (1.3) | 0.03 | 197 (0.6) | 25  (1.29) | <0.001 | 135  (0.6) | 19  (1.5) | <0.001 | 3  (1.4) | 0  (0.0) | 0.553 |
| COVID | 0  (0.0) | 0  (0.0) | 1 | 0  (0.0) | 0  (0.0) | 1 | 0  (0.0) | 0  (0.0) | 1 | 249  (1.1) | 56  (4.4) | <0.001 | 36  (17.4) | 3  (12.5) | 0.545 |
| **Clinical Evolution** |  |  |  |  |  |  |  |  |  |  |  |  |  |  |  |
| Influenza pneumonia* | 1937 (9.2) | 245 (17.3) | <0.001 | 5574 (13.6) | 666 (25.6) | <0.001 | 5799 (17.1) | 594 (28.9) | <0.001 | 4965  (21.1) | 450  (35.4) | <0.001 | 23  (11.1) | 7  (29.2) | 0.022 |
| ICU admission | 1106 (5.4) | 267 (19.7) | <0.001 | 2401 (5.9) | 661 (25.4) | <0.001 | 2238 (6.6) | 526 (26.1) | <0.001 | 1694  (7.2) | 382  (30.1) | <0.001 | 22  (10.7) | 7  (29.2) | 0.019 |

Notes. Abbreviation: ICU, intensive care unit; J09, Influenza due to certain identified influenza viruses; J10, Influenza due to other identified influenza virus; J11, Influenza due to unidentified influenza virus; *J10.9, pneumonia influenza
