# Supplementary material for: Impact of influenza related hospitalization in Spain: characteristics and risk factor of mortality during five influenza seasons (2016 to 2021)
Source: Front Public Health. 2024 Apr 2;12:1360372. doi: 10.3389/fpubh.2024.1360372 (PMC11018950; doi:10.3389/fpubh.2024.1360372)
Supplement: Supplementary file 4 [file Table_4.DOCX]

**Table 4S. Economic burden of influenza hospitalization per age‐group and per year, 2016‐2021, Spain**

| **Age-group** | **2016-2017** | **2017-2018** | **2018-2019** | **2019-2020** | **2020-2021** | **Total** |
| --- | --- | --- | --- | --- | --- | --- |
| **Hospitalization cost, total in euros** |  |  |  |  |  |  |
| 20y | 6222990 | 12811953 | 12192961 | 16730506 | 367617 | 48326028 |
| 20-39y | 3146007 | 6659377 | 6765538 | 8361985 | 114708 | 25047615 |
| 40-59y | 11751354 | 31630419 | 28456074 | 27289857 | 546239 | 99673943 |
| 60-79y | 33922752 | 71410946 | 61250518 | 48261592 | 785021 | 215630828 |
| ≥ 80y | 36084729 | 57001612 | 45876981 | 26605112 | 416186 | 165984621 |
| Overall | 91127832 | 179514307 | 155542073 | 127249052 | 2229771 | 554663035 |
| **Hospitalization, median (IQR)** |  |  |  |  |  |  |
| 20y | 2437.59  (1756.04-3087.80) | 2437.59  (1577.01-3052.19) | 2437.59  (1577.01-3052.19) | 2754.84  (1974.59-3737.45) | 3366.55  (2754.84-5054.09) | 2504.86  (1921.79-3222.28) |
| 20-39y | 2482.30  (2060.63-3295.19 | 2085.15  (2627.50-3440.36) | 2627.50  (2085.15-3440.36) | 3179.90  (2398.23-3997.79 | 3179.90  (2420.43-3585.02) | 2754.84  (2161.30-3676.74) |
| 40-59y | 3252.80  (2437.59-3664.13) | 3243.38  (2506.36-3729.83) | 3243.38  (2506.36-3729.83) | 3997.79  (3179.90-4780.92) | 4531.38  (3191.29-6405.67) | 3252.80  (2527.48-4221.82) |
| 60-79y | 3252.80  (2437.59-3650.67) | 3243.38  (2506.36-3508.38) | 3243.38  (2506.36-3508.38) | 3997.79  (3179.90-5354.66) | 4621.01  (3737.45-7356.76) | 3252.80  (2527.48-3997.79) |
| ≥ 80y | 3252.80  (2437.59-3504.59) | 3243.38  (2506.36-3508.38) | 3243.38  (2506.36-3508.38) | 3997.79  (3179.90-4408.41) | 4079.20  (3424.93-6274.03) | 3243.38  (2527.48-3604.35) |
| Overall | 3252.80  (2437.59-3504.59) | 3226.25  (2506.36-3508.38) | 3289.26  (2527.48-3604.35) | 3840.48  (3179.90- 4408.41) | 4079.20  (3179.90- 6405.67) | 3243.38  (2506.36-3737.45) |
